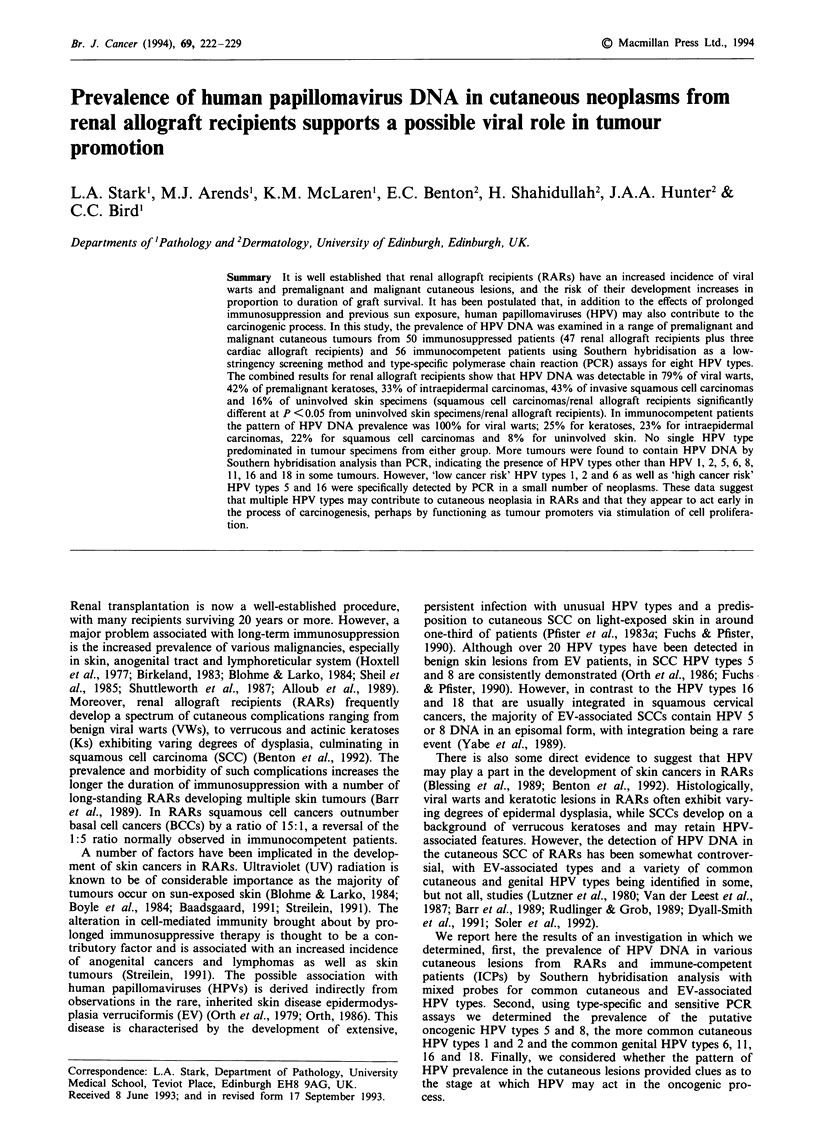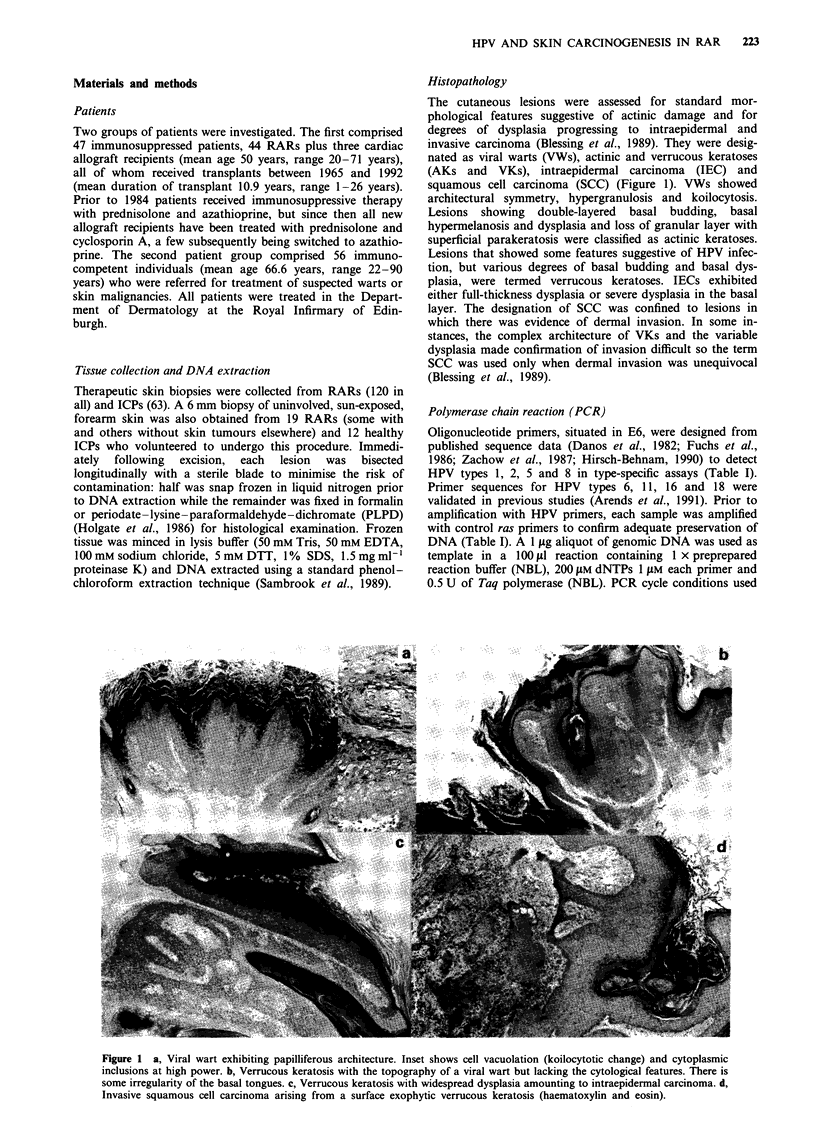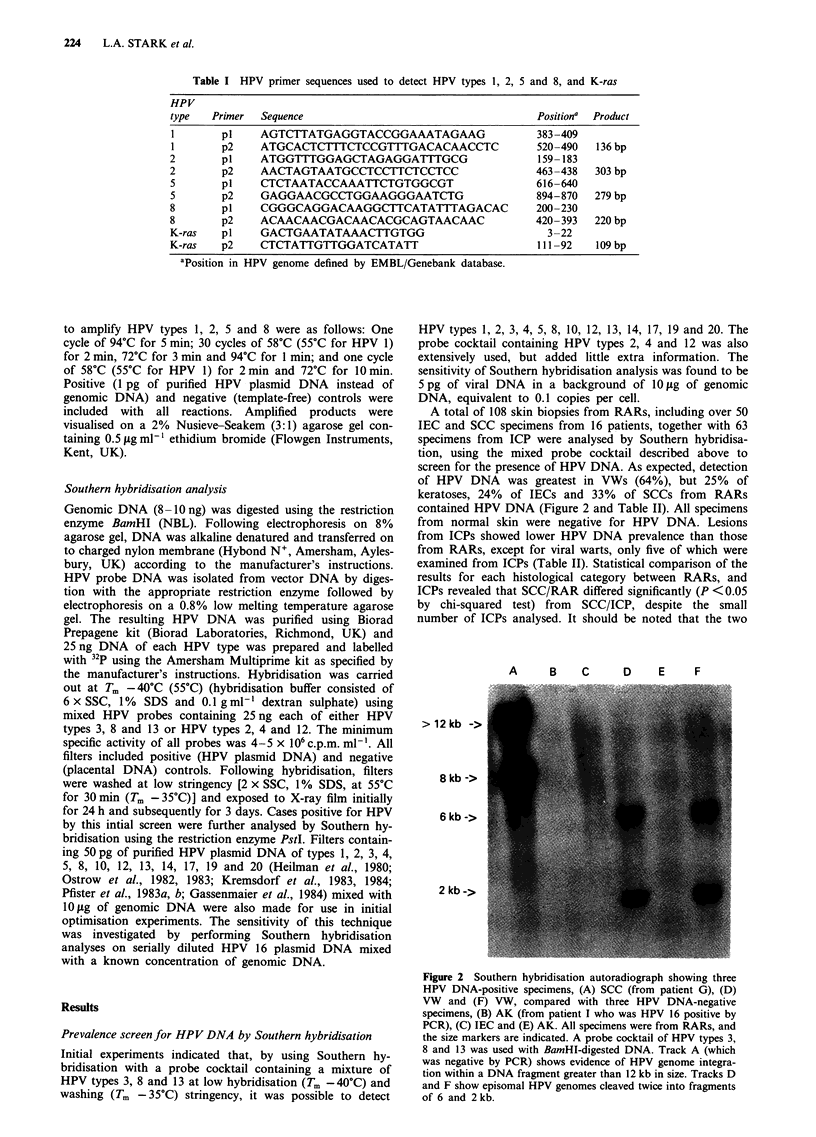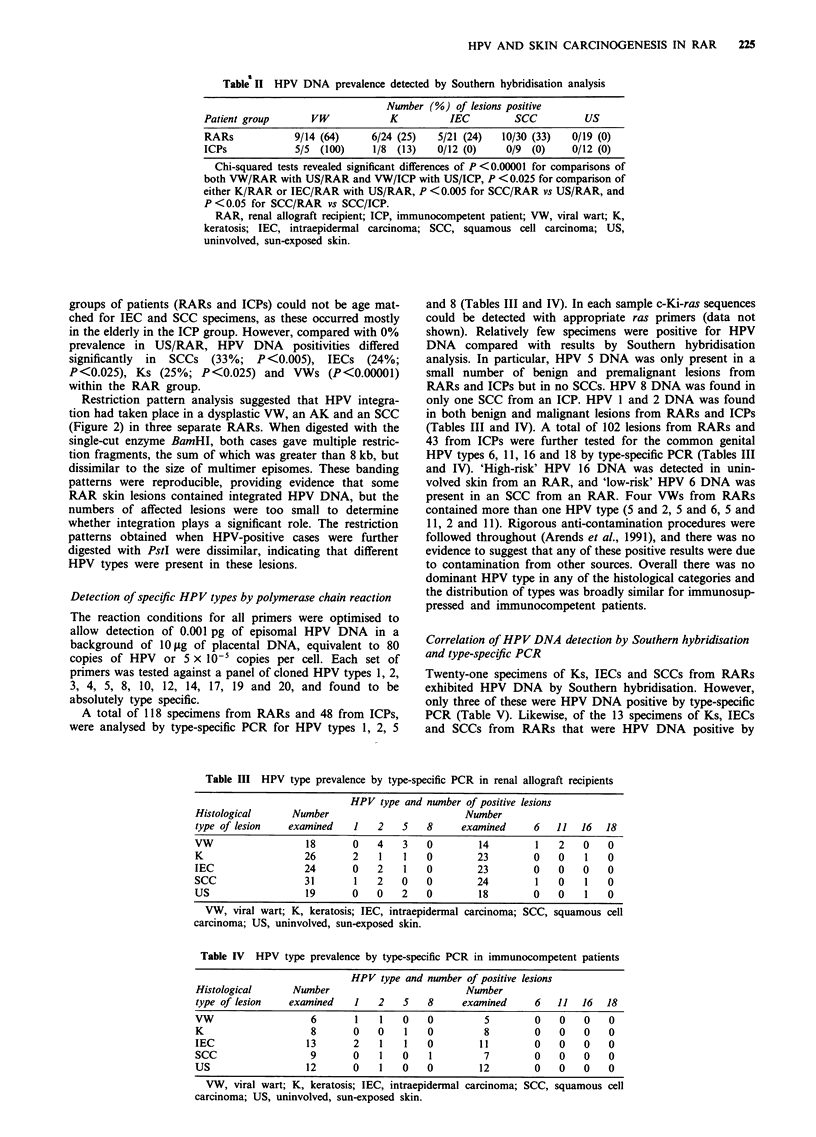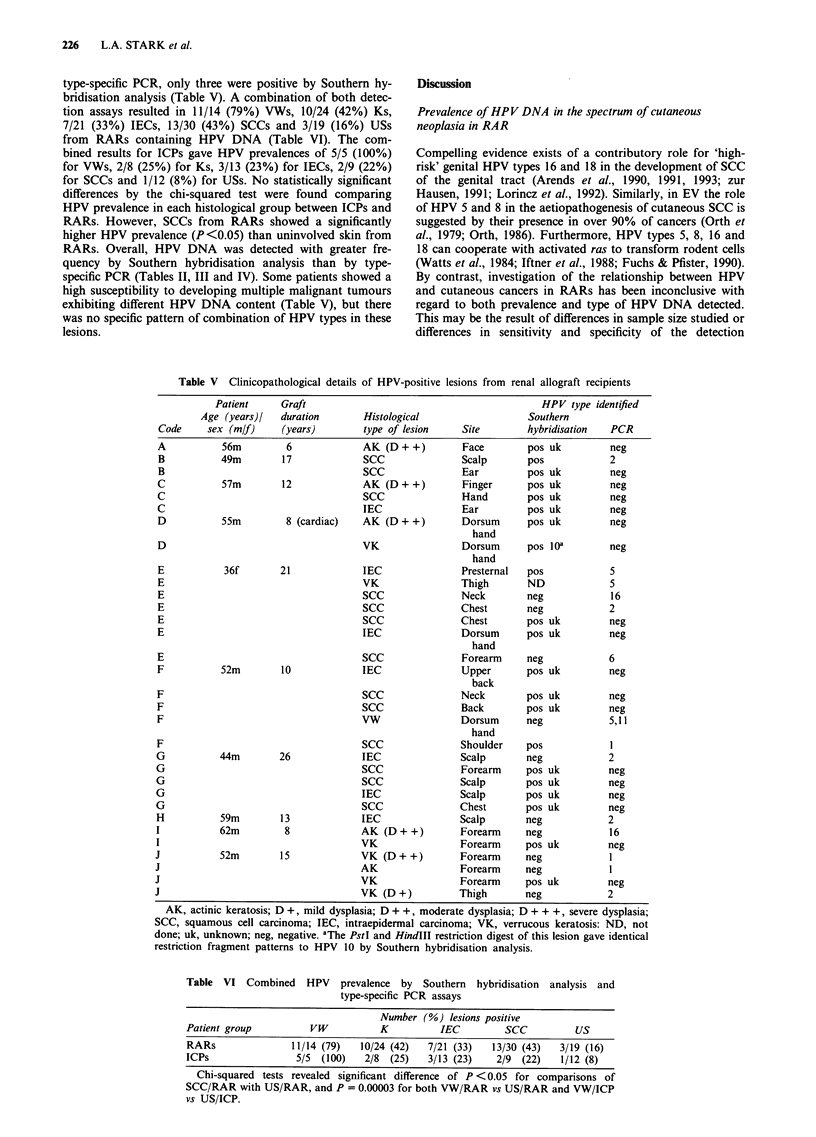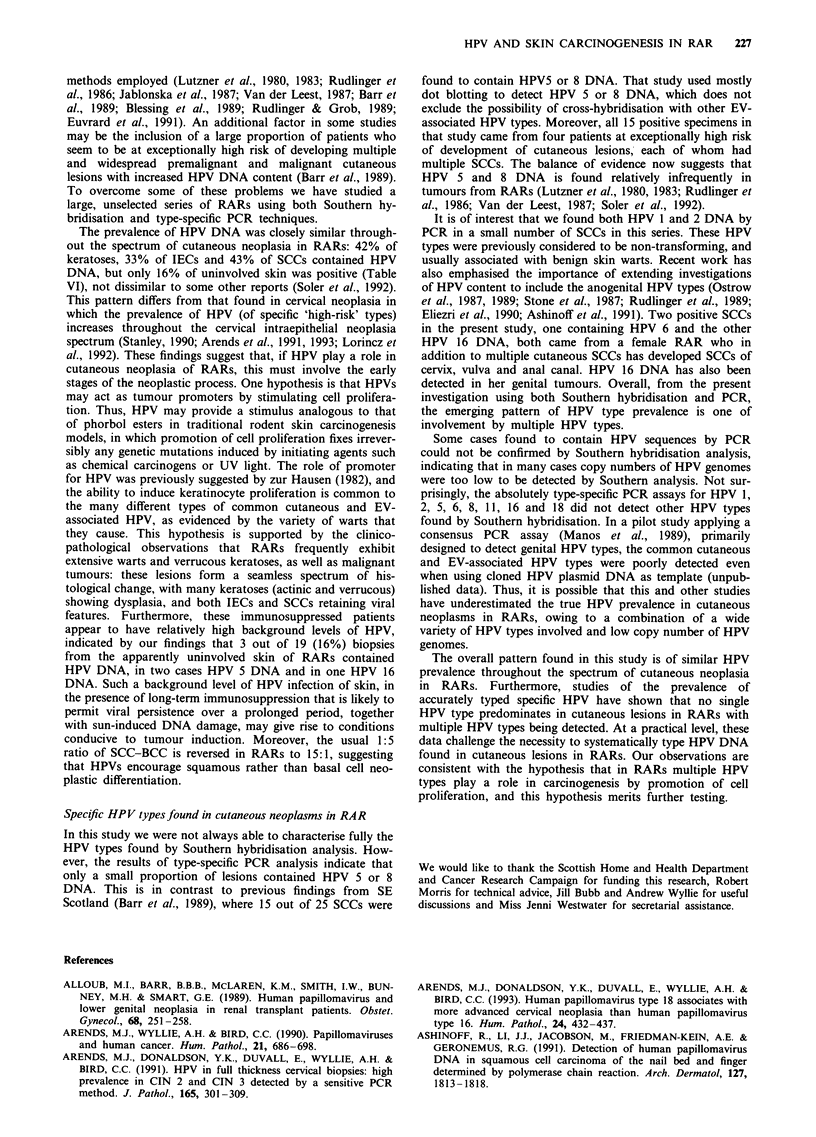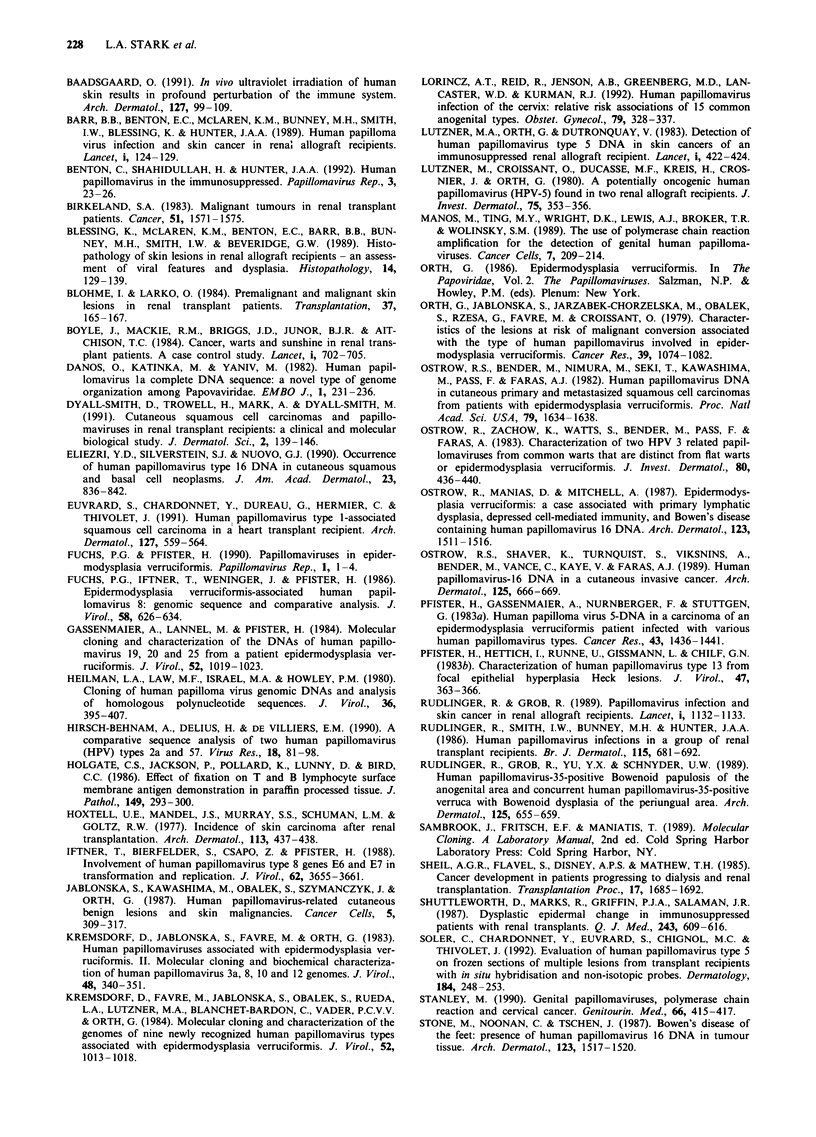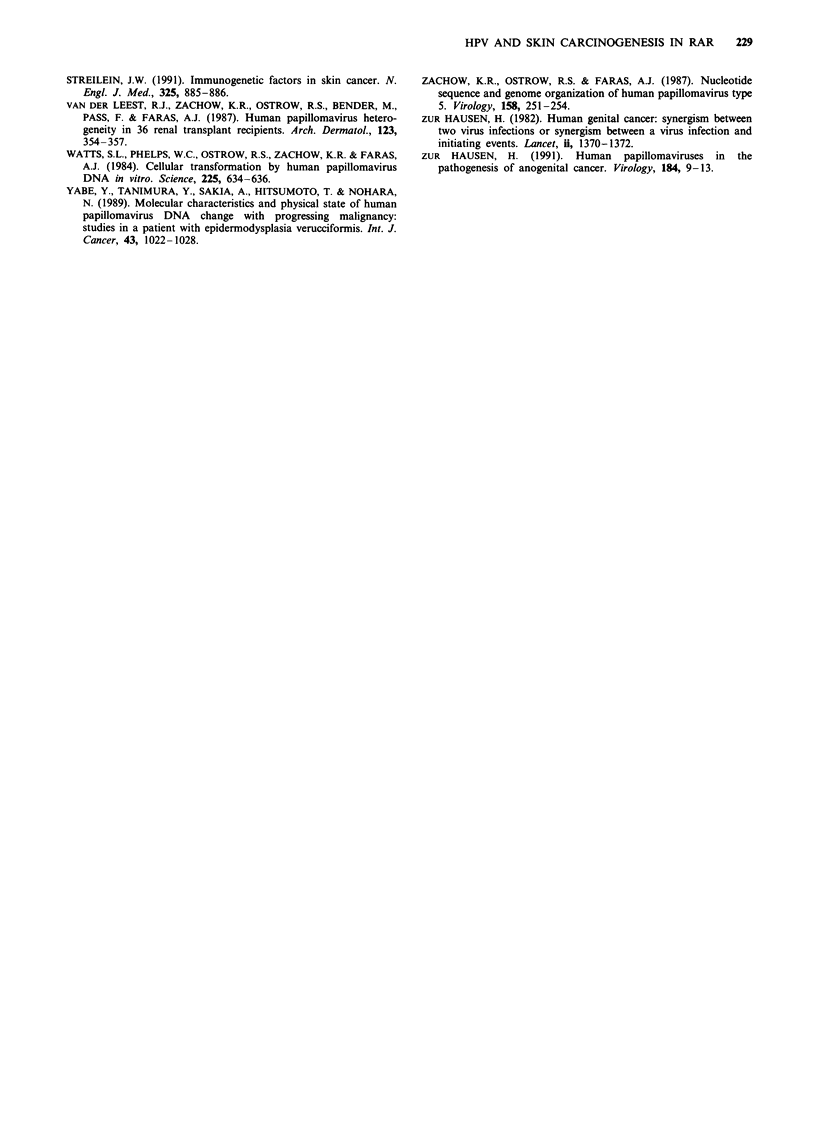# Prevalence of human papillomavirus DNA in cutaneous neoplasms from renal allograft recipients supports a possible viral role in tumour promotion.

**DOI:** 10.1038/bjc.1994.43

**Published:** 1994-02

**Authors:** L. A. Stark, M. J. Arends, K. M. McLaren, E. C. Benton, H. Shahidullah, J. A. Hunter, C. C. Bird

**Affiliations:** Department of Pathology, University of Edinburgh, UK.

## Abstract

**Images:**


Br. J. Cancer (1994), 69, 222-229 © Macmillan Press Ltd., 1994

### Prevalence of human papillomavirus DNA in cutaneous neoplasms from renal allograft recipients supports a possible viral role in tumour promotion

L.A. Stark[1], M.J. Arends[1], K.M. McLaren[1], E.C. Benton[2], H. Shahidullah[2], J.A.A. Hunter[2] & C.C. Bird[1]

*Departments of [1]Pathology and [2]Dermatology, University of Edinburgh, Edinburgh, UK.*

**Summary** It is well established that renal allograpft recipients (RARs) have an increased incidence of viral warts and premalignant and malignant cutaneous lesions, and the risk of their development increases in proportion to duration of graft survival. It has been postulated that, in addition to the effects of prolonged immunosuppression and previous sun exposure, human papillomaviruses (HPV) may also contribute to the carcinogenic process. In this study, the prevalence of HPV DNA was examined in a range of premalignant and malignant cutaneous tumours from 50 immunosuppressed patients (47 renal allograft recipients plus three cardiac allograft recipients) and 56 immunocompetent patients using Southern hybridisation as a low-stringency screening method and type-specific polymerase chain reaction (PCR) assays for eight HPV types. The combined results for renal allograft recipients show that HPV DNA was detectable in 79% of viral warts, 42% of premalignant keratoses, 33% of intraepidermal carcinomas, 43% of invasive squamous cell carcinomas and 16% of uninvolved skin specimens (squamous cell carcinomas/renal allograft recipients significantly different at $P<0.05$ from uninvolved skin specimens/renal allograft recipients). In immunocompetent patients the pattern of HPV DNA prevalence was 100% for viral warts; 25% for keratoses, 23% for intraepidermal carcinomas, 22% for squamous cell carcinomas and 8% for uninvolved skin. No single HPV type predominated in tumour specimens from either group. More tumours were found to contain HPV DNA by Southern hybridisation analysis than PCR, indicating the presence of HPV types other than HPV 1, 2, 5, 6, 8, 11, 16 and 18 in some tumours. However, 'low cancer risk' HPV types 1, 2 and 6 as well as 'high cancer risk' HPV types 5 and 16 were specifically detected by PCR in a small number of neoplasms. These data suggest that multiple HPV types may contribute to cutaneous neoplasia in RARs and that they appear to act early in the process of carcinogenesis, perhaps by functioning as tumour promoters via stimulation of cell proliferation.

Renal transplantation is now a well-established procedure, with many recipients surviving 20 years or more. However, a major problem associated with long-term immunosuppression is the increased prevalence of various malignancies, especially in skin, anogenital tract and lymphoreticular system (Hoxtell *et al.*, 1977; Birkeland, 1983; Blohme & Larko, 1984; Sheil *et al.*, 1985; Shuttleworth *et al.*, 1987; Alloub *et al.*, 1989). Moreover, renal allograft recipients (RARs) frequently develop a spectrum of cutaneous complications ranging from benign viral warts (VWs), to verrucous and actinic keratoses (Ks) exhibiting varing degrees of dysplasia, culminating in squamous cell carcinoma (SCC) (Benton *et al.*, 1992). The prevalence and morbidity of such complications increases the longer the duration of immunosuppression with a number of long-standing RARs developing multiple skin tumours (Barr *et al.*, 1989). In RARs squamous cell cancers outnumber basal cell cancers (BCCs) by a ratio of 15:1, a reversal of the 1:5 ratio normally observed in immunocompetent patients.

A number of factors have been implicated in the development of skin cancers in RARs. Ultraviolet (UV) radiation is known to be of considerable importance as the majority of tumours occur on sun-exposed skin (Blohme & Larko, 1984; Boyle *et al.*, 1984; Baadsgaard, 1991; Streilein, 1991). The alteration in cell-mediated immunity brought about by prolonged immunosuppressive therapy is thought to be a contributory factor and is associated with an increased incidence of anogenital cancers and lymphomas as well as skin tumours (Streilein, 1991). The possible association with human papillomaviruses (HPVs) is derived indirectly from observations in the rare, inherited skin disease epidermodysplasia verruciformis (EV) (Orth *et al.*, 1979; Orth, 1986). This disease is characterised by the development of extensive, persistent infection with unusual HPV types and a predisposition to cutaneous SCC on light-exposed skin in around one-third of patients (Pfister *et al.*, 1983*a*; Fuchs & Pfister, 1990). Although over 20 HPV types have been detected in benign skin lesions from EV patients, in SCC HPV types 5 and 8 are consistently demonstrated (Orth *et al.*, 1986; Fuchs & Pfister, 1990). However, in contrast to the HPV types 16 and 18 that are usually integrated in squamous cervical cancers, the majority of EV-associated SCCs contain HPV 5 or 8 DNA in an episomal form, with integration being a rare event (Yabe *et al.*, 1989).

There is also some direct evidence to suggest that HPV may play a part in the development of skin cancers in RARs (Blessing *et al.*, 1989; Benton *et al.*, 1992). Histologically, viral warts and keratotic lesions in RARs often exhibit varying degrees of epidermal dysplasia, while SCCs develop on a background of verrucous keratoses and may retain HPV-associated features. However, the detection of HPV DNA in the cutaneous SCC of RARs has been somewhat controversial, with EV-associated types and a variety of common cutaneous and genital HPV types being identified in some, but not all, studies (Lutzner *et al.*, 1980; Van der Leest *et al.*, 1987; Barr *et al.*, 1989; Rudlinger & Grob, 1989; Dyall-Smith *et al.*, 1991; Soler *et al.*, 1992).

We report here the results of an investigation in which we determined, first, the prevalence of HPV DNA in various cutaneous lesions from RARs and immune-competent patients (ICPs) by Southern hybridisation analysis with mixed probes for common cutaneous and EV-associated HPV types. Second, using type-specific and sensitive PCR assays we determined the prevalence of the putative oncogenic HPV types 5 and 8, the more common cutaneous HPV types 1 and 2 and the common genital HPV types 6, 11, 16 and 18. Finally, we considered whether the pattern of HPV prevalence in the cutaneous lesions provided clues as to the stage at which HPV may act in the oncogenic process.

Correspondence: L.A. Stark, Department of Pathology, University Medical School, Teviot Place, Edinburgh EH8 9AG, UK.
Received 8 June 1993; and in revised form 17 September 1993.

### Materials and methods

### *Patients*

Two groups of patients were investigated. The first comprised 47 immunosuppressed patients, 44 RARs plus three cardiac allograft recipients (mean age 50 years, range 20–71 years), all of whom received transplants between 1965 and 1992 (mean duration of transplant 10.9 years, range 1–26 years). Prior to 1984 patients received immunosuppressive therapy with prednisolone and azathioprine, but since then all new allograft recipients have been treated with prednisolone and cyclosporin A, a few subsequently being switched to azathioprine. The second patient group comprised 56 immunocompetent individuals (mean age 66.6 years, range 22–90 years) who were referred for treatment of suspected warts or skin malignancies. All patients were treated in the Department of Dermatology at the Royal Infirmary of Edinburgh.

### *Tissue collection and DNA extraction*

Therapeutic skin biopsies were collected from RARs (120 in all) and ICPs (63). A 6 mm biopsy of uninvolved, sun-exposed, forearm skin was also obtained from 19 RARs (some with and others without skin tumours elsewhere) and 12 healthy ICPs who volunteered to undergo this procedure. Immediately following excision, each lesion was bisected longitudinally with a sterile blade to minimise the risk of contamination: half was snap frozen in liquid nitrogen prior to DNA extraction while the remainder was fixed in formalin or periodate–lysine–paraformaldehyde–dichromate (PLPD) (Holgate *et al.*, 1986) for histological examination. Frozen tissue was minced in lysis buffer (50 mM Tris, 50 mM EDTA, 100 mM sodium chloride, 5 mM DTT, 1% SDS, 1.5 mg $ml^{-1}$ proteinase K) and DNA extracted using a standard phenol–chloroform extraction technique (Sambrook *et al.*, 1989).

### *Histopathology*

The cutaneous lesions were assessed for standard morphological features suggestive of actinic damage and for degrees of dysplasia progressing to intraepidermal and invasive carcinoma (Blessing *et al.*, 1989). They were designated as viral warts (VWs), actinic and verrucous keratoses (AKs and VKs), intraepidermal carcinoma (IEC) and squamous cell carcinoma (SCC) (Figure 1). VWs showed architectural symmetry, hypergranulosis and koilocytosis. Lesions showing double-layered basal budding, basal hypermelanosis and dysplasia and loss of granular layer with superficial parakeratosis were classified as actinic keratoses. Lesions that showed some features suggestive of HPV infection, but various degrees of basal budding and basal dysplasia, were termed verrucous keratoses. IECs exhibited either full-thickness dysplasia or severe dysplasia in the basal layer. The designation of SCC was confined to lesions in which there was evidence of dermal invasion. In some instances, the complex architecture of VKs and the variable dysplasia made confirmation of invasion difficult so the term SCC was used only when dermal invasion was unequivocal (Blessing *et al.*, 1989).

### *Polymerase chain reaction (PCR)*

Oligonucleotide primers, situated in E6, were designed from published sequence data (Danos *et al.*, 1982; Fuchs *et al.*, 1986; Zachow *et al.*, 1987; Hirsch-Behnam, 1990) to detect HPV types 1, 2, 5 and 8 in type-specific assays (Table I). Primer sequences for HPV types 6, 11, 16 and 18 were validated in previous studies (Arends *et al.*, 1991). Prior to amplification with HPV primers, each sample was amplified with control *ras* primers to confirm adequate preservation of DNA (Table I). A 1 μg aliquot of genomic DNA was used as template in a 100 μl reaction containing 1 × preprepared reaction buffer (NBL), 200 μM dNTPs 1 μM each primer and 0.5 U of *Taq* polymerase (NBL). PCR cycle conditions used

**Figure 1** **a**, Viral wart exhibiting papilliferous architecture. Inset shows cell vacuolation (koilocytotic change) and cytoplasmic inclusions at high power. **b**, Verrucous keratosis with the topography of a viral wart but lacking the cytological features. There is some irregularity of the basal tongues. **c**, Verrucous keratosis with widespread dysplasia amounting to intraepidermal carcinoma. **d**, Invasive squamous cell carcinoma arising from a surface exophytic verrucous keratosis (haematoxylin and eosin).

**Table I** HPV primer sequences used to detect HPV types 1, 2, 5 and 8, and K-*ras*

| *HPV type* | *Primer* | *Sequence* | *Position*[a] | *Product* |
|---|---|---|---|---|
| 1 | p1 | AGTCTTATGAGGTACCGGAAATAGAAG | 383–409 | |
| 1 | p2 | ATGCACTCTTTCTCCGTTTGACACAACCTC | 520–490 | 136 bp |
| 2 | p1 | ATGGTTTGGAGCTAGAGGATTTGCG | 159–183 | |
| 2 | p2 | AACTAGTAATGCCTCCTTCTCCTCC | 463–438 | 303 bp |
| 5 | p1 | CTCTAATACCAAATTCTGTGGCGT | 616–640 | |
| 5 | p2 | GAGGAACGCCTGGAAGGGAATCTG | 894–870 | 279 bp |
| 8 | p1 | CGGGCAGGACAAGGCTTCATATTTAGACAC | 200–230 | |
| 8 | p2 | ACAACAACGACAACACGCAGTAACAAC | 420–393 | 220 bp |
| K-*ras* | p1 | GACTGAATATAAACTTGTGG | 3–22 | |
| K-*ras* | p2 | CTCTATTGTTGGATCATATT | 111–92 | 109 bp |

[a]Position in HPV genome defined by EMBL/Genebank database.

to amplify HPV types 1, 2, 5 and 8 were as follows: One cycle of 94°C for 5 min; 30 cycles of 58°C (55°C for HPV 1) for 2 min, 72°C for 3 min and 94°C for 1 min; and one cycle of 58°C (55°C for HPV 1) for 2 min and 72°C for 10 min. Positive (1 pg of purified HPV plasmid DNA instead of genomic DNA) and negative (template-free) controls were included with all reactions. Amplified products were visualised on a 2% Nusieve–Seakem (3:1) agarose gel containing 0.5 $\mu$g ml$^{-1}$ ethidium bromide (Flowgen Instruments, Kent, UK).

*Southern hybridisation analysis*

Genomic DNA (8–10 ng) was digested using the restriction enzyme *Bam*HI (NBL). Following electrophoresis on 8% agarose gel, DNA was alkaline denatured and transferred on to charged nylon membrane (Hybond N$^+$, Amersham, Aylesbury, UK) according to the manufacturer's instructions. HPV probe DNA was isolated from vector DNA by digestion with the appropriate restriction enzyme followed by electrophoresis on a 0.8% low melting temperature agarose gel. The resulting HPV DNA was purified using Biorad Prepagene kit (Biorad Laboratories, Richmond, UK) and 25 ng DNA of each HPV type was prepared and labelled with $^{32}$P using the Amersham Multiprime kit as specified by the manufacturer's instructions. Hybridisation was carried out at $T_m$ −40°C (55°C) (hybridisation buffer consisted of 6 × SSC, 1% SDS and 0.1 g ml$^{-1}$ dextran sulphate) using mixed HPV probes containing 25 ng each of either HPV types 3, 8 and 13 or HPV types 2, 4 and 12. The minimum specific activity of all probes was 4–5 × 10$^6$ c.p.m. ml$^{-1}$. All filters included positive (HPV plasmid DNA) and negative (placental DNA) controls. Following hybridisation, filters were washed at low stringency [2 × SSC, 1% SDS, at 55°C for 30 min ($T_m$ −35°C)] and exposed to X-ray film initially for 24 h and subsequently for 3 days. Cases positive for HPV by this intial screen were further analysed by Southern hybridisation using the restriction enzyme *Pst*I. Filters containing 50 pg of purified HPV plasmid DNA of types 1, 2, 3, 4, 5, 8, 10, 12, 13, 14, 17, 19 and 20 (Heilman *et al.*, 1980; Ostrow *et al.*, 1982, 1983; Kremsdorf *et al.*, 1983, 1984; Pfister *et al.*, 1983*a*, *b*; Gassenmaier *et al.*, 1984) mixed with 10 $\mu$g of genomic DNA were also made for use in initial optimisation experiments. The sensitivity of this technique was investigated by performing Southern hybridisation analyses on serially diluted HPV 16 plasmid DNA mixed with a known concentration of genomic DNA.

### Results

*Prevalence screen for HPV DNA by Southern hybridisation*

Initial experiments indicated that, by using Southern hybridisation with a probe cocktail containing a mixture of HPV types 3, 8 and 13 at low hybridisation ($T_m$ −40°C) and washing ($T_m$ −35°C) stringency, it was possible to detect HPV types 1, 2, 3, 4, 5, 8, 10, 12, 13, 14, 17, 19 and 20. The probe cocktail containing HPV types 2, 4 and 12 was also extensively used, but added little extra information. The sensitivity of Southern hybridisation analysis was found to be 5 pg of viral DNA in a background of 10 $\mu$g of genomic DNA, equivalent to 0.1 copies per cell.

A total of 108 skin biopsies from RARs, including over 50 IEC and SCC specimens from 16 patients, together with 63 specimens from ICP were analysed by Southern hybridisation, using the mixed probe cocktail described above to screen for the presence of HPV DNA. As expected, detection of HPV DNA was greatest in VWs (64%), but 25% of keratoses, 24% of IECs and 33% of SCCs from RARs contained HPV DNA (Figure 2 and Table II). All specimens from normal skin were negative for HPV DNA. Lesions from ICPs showed lower HPV DNA prevalence than those from RARs, except for viral warts, only five of which were examined from ICPs (Table II). Statistical comparison of the results for each histological category between RARs, and ICPs revealed that SCC/RAR differed significantly ($P < 0.05$ by chi-squared test) from SCC/ICP, despite the small number of ICPs analysed. It should be noted that the two

**Figure 2** Southern hybridisation autoradiograph showing three HPV DNA-positive specimens, (A) SCC (from patient G), (D) VW and (F) VW, compared with three HPV DNA-negative specimens, (B) AK (from patient I who was HPV 16 positive by PCR), (C) IEC and (E) AK. All specimens were from RARs, and the size markers are indicated. A probe cocktail of HPV types 3, 8 and 13 was used with *Bam*HI-digested DNA. Track A (which was negative by PCR) shows evidence of HPV genome integration within a DNA fragment greater than 12 kb in size. Tracks D and F show episomal HPV genomes cleaved twice into fragments of 6 and 2 kb.

**Table II** HPV DNA prevalence detected by Southern hybridisation analysis

| Patient group | *VW* | *K* | *IEC* | *SCC* | *US* |
|---|---|---|---|---|---|
| | *Number (%) of lesions positive* | | | | |
| RARs | 9/14 (64) | 6/24 (25) | 5/21 (24) | 10/30 (33) | 0/19 (0) |
| ICPs | 5/5 (100) | 1/8 (13) | 0/12 (0) | 0/9 (0) | 0/12 (0) |

Chi-squared tests revealed significant differences of $P<0.00001$ for comparisons of both VW/RAR with US/RAR and VW/ICP with US/ICP, $P<0.025$ for comparison of either K/RAR or IEC/RAR with US/RAR, $P<0.005$ for SCC/RAR *vs* US/RAR, and $P<0.05$ for SCC/RAR *vs* SCC/ICP.

RAR, renal allograft recipient; ICP, immunocompetent patient; VW, viral wart; K, keratosis; IEC, intraepidermal carcinoma; SCC, squamous cell carcinoma; US, uninvolved, sun-exposed skin.

groups of patients (RARs and ICPs) could not be age matched for IEC and SCC specimens, as these occurred mostly in the elderly in the ICP group. However, compared with 0% prevalence in US/RAR, HPV DNA positivities differed significantly in SCCs (33%; $P<0.005$), IECs (24%; $P<0.025$), Ks (25%; $P<0.025$) and VWs ($P<0.00001$) within the RAR group.

Restriction pattern analysis suggested that HPV integration had taken place in a dysplastic VW, an AK and an SCC (Figure 2) in three separate RARs. When digested with the single-cut enzyme *Bam*HI, both cases gave multiple restriction fragments, the sum of which was greater than 8 kb, but dissimilar to the size of multimer episomes. These banding patterns were reproducible, providing evidence that some RAR skin lesions contained integrated HPV DNA, but the numbers of affected lesions were too small to determine whether integration plays a significant role. The restriction patterns obtained when HPV-positive cases were further digested with *Pst*I were dissimilar, indicating that different HPV types were present in these lesions.

### *Detection of specific HPV types by polymerase chain reaction*

The reaction conditions for all primers were optimised to allow detection of 0.001 pg of episomal HPV DNA in a background of 10 μg of placental DNA, equivalent to 80 copies of HPV or $5 \times 10^{-5}$ copies per cell. Each set of primers was tested against a panel of cloned HPV types 1, 2, 3, 4, 5, 8, 10, 12, 14, 17, 19 and 20, and found to be absolutely type specific.

A total of 118 specimens from RARs and 48 from ICPs, were analysed by type-specific PCR for HPV types 1, 2, 5 and 8 (Tables III and IV). In each sample c-Ki-*ras* sequences could be detected with appropriate *ras* primers (data not shown). Relatively few specimens were positive for HPV DNA compared with results by Southern hybridisation analysis. In particular, HPV 5 DNA was only present in a small number of benign and premalignant lesions from RARs and ICPs but in no SCCs. HPV 8 DNA was found in only one SCC from an ICP. HPV 1 and 2 DNA was found in both benign and malignant lesions from RARs and ICPs (Tables III and IV). A total of 102 lesions from RARs and 43 from ICPs were further tested for the common genital HPV types 6, 11, 16 and 18 by type-specific PCR (Tables III and IV). 'High-risk' HPV 16 DNA was detected in uninvolved skin from an RAR, and 'low-risk' HPV 6 DNA was present in an SCC from an RAR. Four VWs from RARs contained more than one HPV type (5 and 2, 5 and 6, 5 and 11, 2 and 11). Rigorous anti-contamination procedures were followed throughout (Arends *et al.*, 1991), and there was no evidence to suggest that any of these positive results were due to contamination from other sources. Overall there was no dominant HPV type in any of the histological categories and the distribution of types was broadly similar for immunosuppressed and immunocompetent patients.

### *Correlation of HPV DNA detection by Southern hybridisation and type-specific PCR*

Twenty-one specimens of Ks, IECs and SCCs from RARs exhibited HPV DNA by Southern hybridisation. However, only three of these were HPV DNA positive by type-specific PCR (Table V). Likewise, of the 13 specimens of Ks, IECs and SCCs from RARs that were HPV DNA positive by

**Table III** HPV type prevalence by type-specific PCR in renal allograft recipients

| *Histological type of lesion* | *Number examined* | *1* | *2* | *5* | *8* | *Number examined* | *6* | *11* | *16* | *18* |
|---|---|---|---|---|---|---|---|---|---|---|
| | | *HPV type and number of positive lesions* | | | | | | | | |
| VW | 18 | 0 | 4 | 3 | 0 | 14 | 1 | 2 | 0 | 0 |
| K | 26 | 2 | 1 | 1 | 0 | 23 | 0 | 0 | 1 | 0 |
| IEC | 24 | 0 | 2 | 1 | 0 | 23 | 0 | 0 | 0 | 0 |
| SCC | 31 | 1 | 2 | 0 | 0 | 24 | 1 | 0 | 1 | 0 |
| US | 19 | 0 | 0 | 2 | 0 | 18 | 0 | 0 | 1 | 0 |

VW, viral wart; K, keratosis; IEC, intraepidermal carcinoma; SCC, squamous cell carcinoma; US, uninvolved, sun-exposed skin.

**Table IV** HPV type prevalence by type-specific PCR in immunocompetent patients

| *Histological type of lesion* | *Number examined* | *1* | *2* | *5* | *8* | *Number examined* | *6* | *11* | *16* | *18* |
|---|---|---|---|---|---|---|---|---|---|---|
| | | *HPV type and number of positive lesions* | | | | | | | | |
| VW | 6 | 1 | 1 | 0 | 0 | 5 | 0 | 0 | 0 | 0 |
| K | 8 | 0 | 0 | 1 | 0 | 8 | 0 | 0 | 0 | 0 |
| IEC | 13 | 2 | 1 | 1 | 0 | 11 | 0 | 0 | 0 | 0 |
| SCC | 9 | 0 | 1 | 0 | 1 | 7 | 0 | 0 | 0 | 0 |
| US | 12 | 0 | 1 | 0 | 0 | 12 | 0 | 0 | 0 | 0 |

VW, viral wart; K, keratosis; IEC, intraepidermal carcinoma; SCC, squamous cell carcinoma; US, uninvolved, sun-exposed skin.

type-specific PCR, only three were positive by Southern hybridisation analysis (Table V). A combination of both detection assays resulted in 11/14 (79%) VWs, 10/24 (42%) Ks, 7/21 (33%) IECs, 13/30 (43%) SCCs and 3/19 (16%) USs from RARs containing HPV DNA (Table VI). The combined results for ICPs gave HPV prevalences of 5/5 (100%) for VWs, 2/8 (25%) for Ks, 3/13 (23%) for IECs, 2/9 (22%) for SCCs and 1/12 (8%) for USs. No statistically significant differences by the chi-squared test were found comparing HPV prevalence in each histological group between ICPs and RARs. However, SCCs from RARs showed a significantly higher HPV prevalence ($P<0.05$) than uninvolved skin from RARs. Overall, HPV DNA was detected with greater frequency by Southern hybridisation analysis than by type-specific PCR (Tables II, III and IV). Some patients showed a high susceptibility to developing multiple malignant tumours exhibiting different HPV DNA content (Table V), but there was no specific pattern of combination of HPV types in these lesions.

### Discussion

*Prevalence of HPV DNA in the spectrum of cutaneous neoplasia in RAR*

Compelling evidence exists of a contributory role for 'high-risk' genital HPV types 16 and 18 in the development of SCC of the genital tract (Arends *et al.*, 1990, 1991, 1993; zur Hausen, 1991; Lorincz *et al.*, 1992). Similarly, in EV the role of HPV 5 and 8 in the aetiopathogenesis of cutaneous SCC is suggested by their presence in over 90% of cancers (Orth *et al.*, 1979; Orth, 1986). Furthermore, HPV types 5, 8, 16 and 18 can cooperate with activated *ras* to transform rodent cells (Watts *et al.*, 1984; Iftner *et al.*, 1988; Fuchs & Pfister, 1990). By contrast, investigation of the relationship between HPV and cutaneous cancers in RARs has been inconclusive with regard to both prevalence and type of HPV DNA detected. This may be the result of differences in sample size studied or differences in sensitivity and specificity of the detection

**Table V** Clinicopathological details of HPV-positive lesions from renal allograft recipients

| Code | Patient Age (years)/ sex (m/f) | Graft duration (years) | Histological type of lesion | Site | HPV type identified Southern hybridisation | PCR |
|---|---|---|---|---|---|---|
| A | 56m | 6 | AK (D++) | Face | pos uk | neg |
| B | 49m | 17 | SCC | Scalp | pos | 2 |
| B | | | SCC | Ear | pos uk | neg |
| C | 57m | 12 | AK (D++) | Finger | pos uk | neg |
| C | | | SCC | Hand | pos uk | neg |
| C | | | IEC | Ear | pos uk | neg |
| D | 55m | 8 (cardiac) | AK (D++) | Dorsum hand | pos uk | neg |
| D | | | VK | Dorsum hand | pos 10[a] | neg |
| E | 36f | 21 | IEC | Presternal | pos | 5 |
| E | | | VK | Thigh | ND | 5 |
| E | | | SCC | Neck | neg | 16 |
| E | | | SCC | Chest | neg | 2 |
| E | | | SCC | Chest | pos uk | neg |
| E | | | IEC | Dorsum hand | pos uk | neg |
| E | | | SCC | Forearm | neg | 6 |
| F | 52m | 10 | IEC | Upper back | pos uk | neg |
| F | | | SCC | Neck | pos uk | neg |
| F | | | SCC | Back | pos uk | neg |
| F | | | VW | Dorsum hand | neg | 5,11 |
| F | | | SCC | Shoulder | pos | 1 |
| G | 44m | 26 | IEC | Scalp | neg | 2 |
| G | | | SCC | Forearm | pos uk | neg |
| G | | | SCC | Scalp | pos uk | neg |
| G | | | IEC | Scalp | pos uk | neg |
| G | | | SCC | Chest | pos uk | neg |
| H | 59m | 13 | IEC | Scalp | neg | 2 |
| I | 62m | 8 | AK (D++) | Forearm | neg | 16 |
| I | | | VK | Forearm | pos uk | neg |
| J | 52m | 15 | VK (D++) | Forearm | neg | 1 |
| J | | | AK | Forearm | neg | 1 |
| J | | | VK | Forearm | pos uk | neg |
| J | | | VK (D+) | Thigh | neg | 2 |

AK, actinic keratosis; D+, mild dysplasia; D++, moderate dysplasia; D+++, severe dysplasia; SCC, squamous cell carcinoma; IEC, intraepidermal carcinoma; VK, verrucous keratosis: ND, not done; uk, unknown; neg, negative. [a]The *Pst*I and *Hind*III restriction digest of this lesion gave identical restriction fragment patterns to HPV 10 by Southern hybridisation analysis.

**Table VI** Combined HPV prevalence by Southern hybridisation analysis and type-specific PCR assays

| Patient group | Number (%) lesions positive VW | K | IEC | SCC | US |
|---|---|---|---|---|---|
| RARs | 11/14 (79) | 10/24 (42) | 7/21 (33) | 13/30 (43) | 3/19 (16) |
| ICPs | 5/5 (100) | 2/8 (25) | 3/13 (23) | 2/9 (22) | 1/12 (8) |

Chi-squared tests revealed significant difference of $P<0.05$ for comparisons of SCC/RAR with US/RAR, and $P=0.00003$ for both VW/RAR *vs* US/RAR and VW/ICP *vs* US/ICP.

methods employed (Lutzner *et al*., 1980, 1983; Rudlinger *et al*., 1986; Jablonska *et al*., 1987; Van der Leest, 1987; Barr *et al*., 1989; Blessing *et al*., 1989; Rudlinger & Grob, 1989; Euvrard *et al*., 1991). An additional factor in some studies may be the inclusion of a large proportion of patients who seem to be at exceptionally high risk of developing multiple and widespread premalignant and malignant cutaneous lesions with increased HPV DNA content (Barr *et al*., 1989). To overcome some of these problems we have studied a large, unselected series of RARs using both Southern hybridisation and type-specific PCR techniques.

The prevalence of HPV DNA was closely similar throughout the spectrum of cutaneous neoplasia in RARs: 42% of keratoses, 33% of IECs and 43% of SCCs contained HPV DNA, but only 16% of uninvolved skin was positive (Table VI), not dissimilar to some other reports (Soler *et al*., 1992). This pattern differs from that found in cervical neoplasia in which the prevalence of HPV (of specific 'high-risk' types) increases throughout the cervical intraepithelial neoplasia spectrum (Stanley, 1990; Arends *et al*., 1991, 1993; Lorincz *et al*., 1992). These findings suggest that, if HPV play a role in cutaneous neoplasia of RARs, this must involve the early stages of the neoplastic process. One hypothesis is that HPVs may act as tumour promoters by stimulating cell proliferation. Thus, HPV may provide a stimulus analogous to that of phorbol esters in traditional rodent skin carcinogenesis models, in which promotion of cell proliferation fixes irreversibly any genetic mutations induced by initiating agents such as chemical carcinogens or UV light. The role of promoter for HPV was previously suggested by zur Hausen (1982), and the ability to induce keratinocyte proliferation is common to the many different types of common cutaneous and EV-associated HPV, as evidenced by the variety of warts that they cause. This hypothesis is supported by the clinicopathological observations that RARs frequently exhibit extensive warts and verrucous keratoses, as well as malignant tumours: these lesions form a seamless spectrum of histological change, with many keratoses (actinic and verrucous) showing dysplasia, and both IECs and SCCs retaining viral features. Furthermore, these immunosuppressed patients appear to have relatively high background levels of HPV, indicated by our findings that 3 out of 19 (16%) biopsies from the apparently uninvolved skin of RARs contained HPV DNA, in two cases HPV 5 DNA and in one HPV 16 DNA. Such a background level of HPV infection of skin, in the presence of long-term immunosuppression that is likely to permit viral persistence over a prolonged period, together with sun-induced DNA damage, may give rise to conditions conducive to tumour induction. Moreover, the usual 1:5 ratio of SCC–BCC is reversed in RARs to 15:1, suggesting that HPVs encourage squamous rather than basal cell neoplastic differentiation.

*Specific HPV types found in cutaneous neoplasms in RAR*

In this study we were not always able to characterise fully the HPV types found by Southern hybridisation analysis. However, the results of type-specific PCR analysis indicate that only a small proportion of lesions contained HPV 5 or 8 DNA. This is in contrast to previous findings from SE Scotland (Barr *et al*., 1989), where 15 out of 25 SCCs were found to contain HPV5 or 8 DNA. That study used mostly dot blotting to detect HPV 5 or 8 DNA, which does not exclude the possibility of cross-hybridisation with other EV-associated HPV types. Moreover, all 15 positive specimens in that study came from four patients at exceptionally high risk of development of cutaneous lesions, each of whom had multiple SCCs. The balance of evidence now suggests that HPV 5 and 8 DNA is found relatively infrequently in tumours from RARs (Lutzner *et al*., 1980, 1983; Rudlinger *et al*., 1986; Van der Leest, 1987; Soler *et al*., 1992).

It is of interest that we found both HPV 1 and 2 DNA by PCR in a small number of SCCs in this series. These HPV types were previously considered to be non-transforming, and usually associated with benign skin warts. Recent work has also emphasised the importance of extending investigations of HPV content to include the anogenital HPV types (Ostrow *et al*., 1987, 1989; Stone *et al*., 1987; Rudlinger *et al*., 1989; Eliezri *et al*., 1990; Ashinoff *et al*., 1991). Two positive SCCs in the present study, one containing HPV 6 and the other HPV 16 DNA, both came from a female RAR who in addition to multiple cutaneous SCCs has developed SCCs of cervix, vulva and anal canal. HPV 16 DNA has also been detected in her genital tumours. Overall, from the present investigation using both Southern hybridisation and PCR, the emerging pattern of HPV type prevalence is one of involvement by multiple HPV types.

Some cases found to contain HPV sequences by PCR could not be confirmed by Southern hybridisation analysis, indicating that in many cases copy numbers of HPV genomes were too low to be detected by Southern analysis. Not surprisingly, the absolutely type-specific PCR assays for HPV 1, 2, 5, 6, 8, 11, 16 and 18 did not detect other HPV types found by Southern hybridisation. In a pilot study applying a consensus PCR assay (Manos *et al*., 1989), primarily designed to detect genital HPV types, the common cutaneous and EV-associated HPV types were poorly detected even when using cloned HPV plasmid DNA as template (unpublished data). Thus, it is possible that this and other studies have underestimated the true HPV prevalence in cutaneous neoplasms in RARs, owing to a combination of a wide variety of HPV types involved and low copy number of HPV genomes.

The overall pattern found in this study is of similar HPV prevalence throughout the spectrum of cutaneous neoplasia in RARs. Furthermore, studies of the prevalence of accurately typed specific HPV have shown that no single HPV type predominates in cutaneous lesions in RARs with multiple HPV types being detected. At a practical level, these data challenge the necessity to systematically type HPV DNA found in cutaneous lesions in RARs. Our observations are consistent with the hypothesis that in RARs multiple HPV types play a role in carcinogenesis by promotion of cell proliferation, and this hypothesis merits further testing.

We would like to thank the Scottish Home and Health Department and Cancer Research Campaign for funding this research, Robert Morris for technical advice, Jill Bubb and Andrew Wyllie for useful discussions and Miss Jenni Westwater for secretarial assistance.

### References

ALLOUB, M.I., BARR, B.B.B., MCLAREN, K.M., SMITH, I.W., BUNNEY, M.H. & SMART, G.E. (1989). Human papillomavirus and lower genital neoplasia in renal transplant patients. *Obstet. Gynecol*., **68**, 251–258.

ARENDS, M.J., WYLLIE, A.H. & BIRD, C.C. (1990). Papillomaviruses and human cancer. *Hum. Pathol*., **21**, 686–698.

ARENDS, M.J., DONALDSON, Y.K., DUVALL, E., WYLLIE, A.H. & BIRD, C.C. (1991). HPV in full thickness cervical biopsies: high prevalence in CIN 2 and CIN 3 detected by a sensitive PCR method. *J. Pathol*., **165**, 301–309.

ARENDS, M.J., DONALDSON, Y.K., DUVALL, E., WYLLIE, A.H. & BIRD, C.C. (1993). Human papillomavirus type 18 associates with more advanced cervical neoplasia than human papillomavirus type 16. *Hum. Pathol*., **24**, 432–437.

ASHINOFF, R., LI, J.J., JACOBSON, M., FRIEDMAN-KEIN, A.E. & GERONEMUS, R.G. (1991). Detection of human papillomavirus DNA in squamous cell carcinoma of the nail bed and finger determined by polymerase chain reaction. *Arch. Dermatol*, **127**, 1813–1818.

BAADSGAARD, O. (1991). *In vivo* ultraviolet irradiation of human skin results in profound perturbation of the immune system. *Arch. Dermatol.*, **127**, 99–109.

BARR, B.B., BENTON, E.C., MCLAREN, K.M., BUNNEY, M.H., SMITH, I.W., BLESSING, K. & HUNTER, J.A.A. (1989). Human papilloma virus infection and skin cancer in renal allograft recipients. *Lancet*, **i**, 124–129.

BENTON, C., SHAHIDULLAH, H. & HUNTER, J.A.A. (1992). Human papillomavirus in the immunosuppressed. *Papillomavirus Rep.*, **3**, 23–26.

BIRKELAND, S.A. (1983). Malignant tumours in renal transplant patients. *Cancer*, **51**, 1571–1575.

BLESSING, K., MCLAREN, K.M., BENTON, E.C., BARR, B.B., BUNNEY, M.H., SMITH, I.W. & BEVERIDGE, G.W. (1989). Histopathology of skin lesions in renal allograft recipients – an assessment of viral features and dysplasia. *Histopathology*, **14**, 129–139.

BLOHME, I. & LARKO, O. (1984). Premalignant and malignant skin lesions in renal transplant patients. *Transplantation*, **37**, 165–167.

BOYLE, J., MACKIE, R.M., BRIGGS, J.D., JUNOR, B.J.R. & AITCHISON, T.C. (1984). Cancer, warts and sunshine in renal transplant patients. A case control study. *Lancet*, **i**, 702–705.

DANOS, O., KATINKA, M. & YANIV, M. (1982). Human papillomavirus 1a complete DNA sequence: a novel type of genome organization among Papovaviridae. *EMBO J.*, **1**, 231–236.

DYALL-SMITH, D., TROWELL, H., MARK, A. & DYALL-SMITH, M. (1991). Cutaneous squamous cell carcinomas and papillomaviruses in renal transplant recipients: a clinical and molecular biological study. *J. Dermatol. Sci.*, **2**, 139–146.

ELIEZRI, Y.D., SILVERSTEIN, S.J. & NUOVO, G.J. (1990). Occurrence of human papillomavirus type 16 DNA in cutaneous squamous and basal cell neoplasms. *J. Am. Acad. Dermatol.*, **23**, 836–842.

EUVRARD, S., CHARDONNET, Y., DUREAU, G., HERMIER, C. & THIVOLET, J. (1991). Human papillomavirus type 1-associated squamous cell carcinoma in a heart transplant recipient. *Arch. Dermatol.*, **127**, 559–564.

FUCHS, P.G. & PFISTER, H. (1990). Papillomaviruses in epidermodysplasia verruciformis. *Papillomavirus Rep.*, **1**, 1–4.

FUCHS, P.G., IFTNER, T., WENINGER, J. & PFISTER, H. (1986). Epidermodysplasia verruciformis-associated human papillomavirus 8: genomic sequence and comparative analysis. *J. Virol.*, **58**, 626–634.

GASSENMAIER, A., LANNEL, M. & PFISTER, H. (1984). Molecular cloning and characterization of the DNAs of human papillomavirus 19, 20 and 25 from a patient epidermodysplasia verruciformis. *J. Virol.*, **52**, 1019–1023.

HEILMAN, L.A., LAW, M.F., ISRAEL, M.A. & HOWLEY, P.M. (1980). Cloning of human papilloma virus genomic DNAs and analysis of homologous polynucleotide sequences. *J. Virol.*, **36**, 395–407.

HIRSCH-BEHNAM, A., DELIUS, H. & DE VILLIERS, E.M. (1990). A comparative sequence analysis of two human papillomavirus (HPV) types 2a and 57. *Virus Res.*, **18**, 81–98.

HOLGATE, C.S., JACKSON, P., POLLARD, K., LUNNY, D. & BIRD, C.C. (1986). Effect of fixation on T and B lymphocyte surface membrane antigen demonstration in paraffin processed tissue. *J. Pathol.*, **149**, 293–300.

HOXTELL, U.E., MANDEL, J.S., MURRAY, S.S., SCHUMAN, L.M. & GOLTZ, R.W. (1977). Incidence of skin carcinoma after renal transplantation. *Arch. Dermatol.*, **113**, 437–438.

IFTNER, T., BIERFELDER, S., CSAPO, Z. & PFISTER, H. (1988). Involvement of human papillomavirus type 8 genes E6 and E7 in transformation and replication. *J. Virol.*, **62**, 3655–3661.

JABLONSKA, S., KAWASHIMA, M., OBALEK, S., SZYMANCZYK, J. & ORTH, G. (1987). Human papillomavirus-related cutaneous benign lesions and skin malignancies. *Cancer Cells*, **5**, 309–317.

KREMSDORF, D., JABLONSKA, S., FAVRE, M. & ORTH, G. (1983). Human papillomaviruses associated with epidermodysplasia verruciformis. II. Molecular cloning and biochemical characterization of human papillomavirus 3a, 8, 10 and 12 genomes. *J. Virol.*, **48**, 340–351.

KREMSDORF, D., FAVRE, M., JABLONSKA, S., OBALEK, S., RUEDA, L.A., LUTZNER, M.A., BLANCHET-BARDON, C., VADER, P.C.V.V. & ORTH, G. (1984). Molecular cloning and characterization of the genomes of nine newly recognized human papillomavirus types associated with epidermodysplasia verruciformis. *J. Virol.*, **52**, 1013–1018.

LORINCZ, A.T., REID, R., JENSON, A.B., GREENBERG, M.D., LANCASTER, W.D. & KURMAN, R.J. (1992). Human papillomavirus infection of the cervix: relative risk associations of 15 common anogenital types. *Obstet. Gynecol.*, **79**, 328–337.

LUTZNER, M.A., ORTH, G. & DUTRONQUAY, V. (1983). Detection of human papillomavirus type 5 DNA in skin cancers of an immunosuppressed renal allograft recipient. *Lancet*, **i**, 422–424.

LUTZNER, M., CROISSANT, O., DUCASSE, M.F., KREIS, H., CROSNIER, J. & ORTH, G. (1980). A potentially oncogenic human papillomavirus (HPV-5) found in two renal allograft recipients. *J. Invest. Dermatol.*, **75**, 353–356.

MANOS, M., TING, M.Y., WRIGHT, D.K., LEWIS, A.J., BROKER, T.R. & WOLINSKY, S.M. (1989). The use of polymerase chain reaction amplification for the detection of genital human papillomaviruses. *Cancer Cells*, **7**, 209–214.

ORTH, G. (1986). Epidermodysplasia verruciformis. In *The Papoviridae*, Vol. 2. *The Papillomaviruses*. Salzman, N.P. & Howley, P.M. (eds). Plenum: New York.

ORTH, G., JABLONSKA, S., JARZABEK-CHORZELSKA, M., OBALEK, S., RZESA, G., FAVRE, M. & CROISSANT, O. (1979). Characteristics of the lesions at risk of malignant conversion associated with the type of human papillomavirus involved in epidermodysplasia verruciformis. *Cancer Res.*, **39**, 1074–1082.

OSTROW, R.S., BENDER, M., NIMURA, M., SEKI, T., KAWASHIMA, M., PASS, F. & FARAS, A.J. (1982). Human papillomavirus DNA in cutaneous primary and metastasized squamous cell carcinomas from patients with epidermodysplasia verruciformis. *Proc. Natl Acad. Sci. USA*, **79**, 1634–1638.

OSTROW, R., ZACHOW, K., WATTS, S., BENDER, M., PASS, F. & FARAS, A. (1983). Characterization of two HPV 3 related papillomaviruses from common warts that are distinct from flat warts or epidermodysplasia verruciformis. *J. Invest. Dermatol.*, **80**, 436–440.

OSTROW, R., MANIAS, D. & MITCHELL, A. (1987). Epidermodysplasia verruciformis: a case associated with primary lymphatic dysplasia, depressed cell-mediated immunity, and Bowen's disease containing human papillomavirus 16 DNA. *Arch. Dermatol.*, **123**, 1511–1516.

OSTROW, R.S., SHAVER, K., TURNQUIST, S., VIKSNINS, A., BENDER, M., VANCE, C., KAYE, V. & FARAS, A.J. (1989). Human papillomavirus-16 DNA in a cutaneous invasive cancer. *Arch. Dermatol.*, **125**, 666–669.

PFISTER, H., GASSENMAIER, A., NURNBERGER, F. & STUTTGEN, G. (1983*a*). Human papilloma virus 5-DNA in a carcinoma of an epidermodysplasia verruciformis patient infected with various human papillomavirus types. *Cancer Res.*, **43**, 1436–1441.

PFISTER, H., HETTICH, I., RUNNE, U., GISSMANN, L. & CHILF, G.N. (1983*b*). Characterization of human papillomavirus type 13 from focal epithelial hyperplasia Heck lesions. *J. Virol.*, **47**, 363–366.

RUDLINGER, R. & GROB, R. (1989). Papillomavirus infection and skin cancer in renal allograft recipients. *Lancet*, **i**, 1132–1133.

RUDLINGER, R., SMITH, I.W., BUNNEY, M.H. & HUNTER, J.A.A. (1986). Human papillomavirus infections in a group of renal transplant recipients. *Br. J. Dermatol.*, **115**, 681–692.

RUDLINGER, R., GROB, R., YU, Y.X. & SCHNYDER, U.W. (1989). Human papillomavirus-35-positive Bowenoid papulosis of the anogenital area and concurrent human papillomavirus-35-positive verruca with Bowenoid dysplasia of the periungual area. *Arch. Dermatol.*, **125**, 655–659.

SAMBROOK, J., FRITSCH, E.F. & MANIATIS, T. (1989). *Molecular Cloning. A Laboratory Manual*, 2nd ed. Cold Spring Harbor Laboratory Press: Cold Spring Harbor, NY.

SHEIL, A.G.R., FLAVEL, S., DISNEY, A.P.S. & MATHEW, T.H. (1985). Cancer development in patients progressing to dialysis and renal transplantation. *Transplantation Proc.*, **17**, 1685–1692.

SHUTTLEWORTH, D., MARKS, R., GRIFFIN, P.J.A., SALAMAN, J.R. (1987). Dysplastic epidermal change in immunosuppressed patients with renal transplants. *Q. J. Med.*, **243**, 609–616.

SOLER, C., CHARDONNET, Y., EUVRARD, S., CHIGNOL, M.C. & THIVOLET, J. (1992). Evaluation of human papillomavirus type 5 on frozen sections of multiple lesions from transplant recipients with *in situ* hybridisation and non-isotopic probes. *Dermatology*, **184**, 248–253.

STANLEY, M. (1990). Genital papillomaviruses, polymerase chain reaction and cervical cancer. *Genitourin. Med.*, **66**, 415–417.

STONE, M., NOONAN, C. & TSCHEN, J. (1987). Bowen's disease of the feet: presence of human papillomavirus 16 DNA in tumour tissue. *Arch. Dermatol.*, **123**, 1517–1520.

STREILEIN, J.W. (1991). Immunogenetic factors in skin cancer. *N. Engl. J. Med.*, **325**, 885–886.

VAN DER LEEST, R.J., ZACHOW, K.R., OSTROW, R.S., BENDER, M., PASS, F. & FARAS, A.J. (1987). Human papillomavirus heterogeneity in 36 renal transplant recipients. *Arch. Dermatol.*, **123**, 354–357.

WATTS, S.L., PHELPS, W.C., OSTROW, R.S., ZACHOW, K.R. & FARAS, A.J. (1984). Cellular transformation by human papillomavirus DNA *in vitro*. *Science*, **225**, 634–636.

YABE, Y., TANIMURA, Y., SAKIA, A., HITSUMOTO, T. & NOHARA, N. (1989). Molecular characteristics and physical state of human papillomavirus DNA change with progressing malignancy: studies in a patient with epidermodysplasia verucciformis. *Int. J. Cancer*, **43**, 1022–1028.

ZACHOW, K.R., OSTROW, R.S. & FARAS, A.J. (1987). Nucleotide sequence and genome organization of human papillomavirus type 5. *Virology*, **158**, 251–254.

ZUR HAUSEN, H. (1982). Human genital cancer: synergism between two virus infections or synergism between a virus infection and initiating events. *Lancet*, **ii**, 1370–1372.

ZUR HAUSEN, H. (1991). Human papillomaviruses in the pathogenesis of anogenital cancer. *Virology*, **184**, 9–13.